# Comparison of the protective actions of N-acetylcysteine, hypotaurine and taurine against acetaminophen-induced hepatotoxicity in the rat

**DOI:** 10.1186/1423-0127-17-S1-S35

**Published:** 2010-08-24

**Authors:** Miteshkumar Acharya, Cesar A Lau-Cam

**Affiliations:** 1 Department of Pharmaceutical Sciences, St. John’s University, College of Pharmacy and Allied Health Professions, 8000 Utopia Parkway, Jamaica, New York 11439, USA

## Abstract

When used in overdoses, acetaminophen (APAP) is a common cause of morbidity and mortality in humans. At present, N-acetylcysteine (NAC) is the antidote of choice for acetaminophen overdoses. Prompt administration of NAC can prevent the deleterious actions of APAP in the liver. In view of the similarities in antioxidant effects demonstrated by NAC, hypotaurine (HYTAU) and taurine (TAU) in this and other our laboratories, the present study was undertaken to compare these compounds for the ability to attenuate plasma and liver biochemical changes associated with a toxic dose of APAP. For this purpose, fasted male Sprague-Dawley rats, 225-250 g in weight, were intraperitoneally treated with APAP (800 mg/kg), NAC, HYTAU or TAU (2.4 mM/kg) followed 30 min later by APAP, or 50% PEG 400 (the vehicle for APAP). At 6 hr after APAP administration, all animals were sacrificed by decapitation and their blood and livers collected. The plasma fractions were analyzed for indices of liver damage (alanine transaminase, aspartate transaminase, lactate dehydrogenase), levels of malondialdehyde (MDA), reduced (GSH) and oxidized (GSSG) glutathione, and activities of glutathione reductase (GR), glutathione S-transferase (GST) and γ-glutamylcisteinyl synthetase (GCS). Suitable liver homogenates were analyzed for the same biochemical parameters as the plasma but indices of liver damage. By itself, APAP increased MDA formation and had a significant lowering influence on the levels of GSH and GSSG, the GSH/GSSH ratio, and the activities of GR, GST and GCS both in the plasma and liver. In addition, APAP promoted the leakage of transaminases and lactate dehydrogenase from the liver into the plasma. Without exceptions, a pretreatment with a sulfur-containing compound led to a significant attenuation of the liver injury and the biochemical changes induced by APAP. Within a narrow range of potency differences, HYTAU appeared to be the most protective and TAU the least. The present results suggest that, irrespective of the differences in structural features and in vitro antioxidant properties that may exist among NAC, TAU and HYTAU, these compounds demonstrate equivalent patterns of protection and, to a certain extent, equipotent protective actions against the toxic actions of APAP in the liver when tested in equimolar doses and under the same conditions in an animal model.

## Background

Therapeutic doses of the analgesic drug acetaminophen (APAP) are readily detoxified by hepatic phase II drug-metabolizing systems mediating glucuronidation and sulfation [1], with a small portion undergoing a cytochrome P-450-mediated bioactivation to the highly reactive electrophilic arylating intermediate N-acetyl-*p*-benzoquinoneimine (NAPQI) [2]. In rats and humans, NAPQI is detoxified principally by conjugation with reduced glutathione (GSH) under spontaneous or glutathione S-transferase (GST)-mediated conditions to the 3-glutathione-S-yl-APAP conjugate [1].

In the event of the intake of an overdose of APAP, the increased production of NAPQI rapidly overwhelms GST, eventually exhausts GSH, UDP-glucuronic acid and inorganic sulfate [3], inhibits GSH synthesis [3,4] and decreases cytosolic GST activity [5]. More importantly, this APAP metabolite is a major cause of hepatocellular damage, centrilobular hepatic necrosis and even fatalities upon entering in adduct formation with liver macromolecules, especially proteins [6].

The hepatotoxicity of APAP is generally recognized to start with the formation of NAPQI and to be related to the oxidative stress that develops as a result of the oxidative capacities of this reactive metabolic product [6-10]. NAPQI is capable of lowering GSH/GSSG ratio by oxidizing the thiol group of GSH and of promoting the formation of interstrand disulfide linkages, interprotein cross links and protein-GSH mixed disulfides by oxidizing cysteine thiol groups in proteins [10]. APAP may also cause hepatotoxicity by mechanisms leading to the formation of reactive oxygen species (ROS), such as superoxide anion (O_2_^-•^), hydrogen peroxide (H_2_O_2_) and hydroxyl radical (HO•), reactive nitrogen species (RNS), such as nitric oxide and peroxynitrite (ONOO^-^), and peroxidation reaction products [6-8,10].

Furthermore, the APAP-associated depletion of the intrahepatic GSH is accompanied by variable alterations in glutathione disulfide (GSSG) contents [11,12] and by reductions in the activities of the antioxidant enzymes glutathione reductase (GR) [13,14], γ-glutamylcysteinyl synthetase (GCS), catalase (CAT), glutathione peroxidase (GPX) and superoxide dismutase (SOD) [14].

Since NAPQI formation, GSH depletion, and the alkylation of proteins, especially in the mitochondrion, are central to the onset of hepatotoxicity by APAP [15], compounds with potential for serving as a source of GSH [3,16-19] or for preventing oxidative reactions [19-24] have been extensively studied for the ability to counteract APAP toxicity. Based on this evidence, the present study was aimed at comparing the hepatoprotective actions of taurine (TAU), a nonprotein sulfur-containing amino acid, and hypotaurine (HYTAU), the immediate metabolic precursor of TAU, with that of N-acetyl-L-cysteine (NAC), an L-cysteine analog that can serve as a substrate for GSH synthesis [16,18,25]. While NAC is regarded as the antidote of choice for APAP overdoses [18] and as an effective antioxidant [26-28], information on the protective actions of TAU in APA-induced acute liver injury appears to be limited to a report indicating that this amino acid can attenuate the leakage of intracellular enzymes from and DNA fragmentation, lipid peroxidation (LPO), apoptosis and necrosis in the hepatocytes of rats receiving a toxic dose of APAP [29]. On the other hand, earlier work from this laboratory has determined that both TAU) and HYTAU can attenuate LPO, preserve the intracellular stores of GSH, and prevent the losses of antioxidant enzyme activities in erythrocytes of rats exposed to an oxidant [30] or affected by type 2 diabetes [31].  In addition, the results of in vitro experiments with free radical generating systems have suggested that HYTAU, because of its sulfinate functionality, is a better antioxidant and radical scavenger than TAU [32]. The purpose of the present study was to compare NAC, HYTAU and TAU for their effects in preventing oxidative stress and, thus, liver injury, when administered in equimolar doses to rats and as a pretreatment to a toxic dose of APAP.

## Methods

### Animals

Male Sprague-Dawley rats, 225-250 g in weight, were purchased from Taconic Farms, Germantown, New York, USA, and housed in a temperature controlled room (21±1°C) with a 12 hr light-12 hr dark cycle; and allowed free access to a standard rat chow and filtered tap water for at least 5 days. The solid food, but not the water, was removed 12 hr prior to an experiment. The study received the approval of the Institutional Animal Care and Use Committee of St. John’s University, and the animals were cared for in accordance with the guidelines established by the United States Department of Agriculture.

### Treatments and samples

The treatment solutions were prepared either in warm 50% PEG 400 (APAP) or in distilled water (NAC, TAU, HYTAU). All the solutions were administered by the intraperitoneal (i.p.) route. A treatment compound was administered as a single, 2.4 mM/kg/2 mL dose, 30 min before APAP (800 mg/kg/2 mL). Control animals received only 50% PEG 400 or only a sulfur-containing compound. The animals were sacrificed by decapitation at 6 hr post-APAP, and their blood samples were promptly delivered into heparinized test tubes for subsequent centrifugation and processing for the plasma fraction. Following surgery of the animals, the livers were exposed and immediately freeze-clamped with metal tongs precooled in liquid nitrogen, and stored in liquid nitrogen until needed.

### Liver homogenates

A liver homogenate for the assay of GSH and GSSG was prepared by mixing a portion of liver sample with 4 times its volume of 25% metaphosphoric acid plus 14 times its volume of PBS pH 8.0, followed by homogenization on ice with the help of a hand held electric blender. The suspension was centrifuged at 5,000 rpm and 4^o^C for 5 min, and the resulting supernatant was stored on ice until needed. For the remaining assays, a liver homogenate was prepared by mixing one part of liver sample with 20 times its volume of 0.01% phenylmethylsulfonyl fluoride in Tris buffer pH 7.0 followed by homogenization with a hand held electric blender while on an ice bath. The suspension was centrifuged at 12,000 rpm and 4°C for 30 min, and the resulting supernatant was stored on ice until needed

### Assay of MDA

The concentration of MDA in the liver and plasma was measured as TBARS using a published colorimetric method [33]. To this effect, an aliquot of liver homogenate (or of plasma) was mixed with a reagent consisting of 15% TCA (w/v)-0.375% TBA (w/v)-0.25 N HCl in a 1:9 ratio (by volume), and heated at 90^o^C for 1 hr. After allowing it to cool to room temperature, the mixture was centrifuged at 2000 x g for 5 min to remove insolubles, and its absorbance read at 535 nm on a spectrophotometer. The level of MDA was derived from a standard curve prepared from serial dilutions of a stock solution of TEP that were treated in an identical manner as the liver samples. The concentration of MDA was expressed as nmol/mg of protein.

### Assay of GSH and GSSG

The concentrations of GSH and GSSG in a liver sample were measured by a fluorometric method that uses OPT as a fluorescent reagent [34]. The method takes advantage of the reaction of GSH with OPT at pH 8 and of GSSG with OPT at pH 12. An aliquot of liver homogenate in 25% metaphosphoric acid (or plasma) was mixed with 9 times its volume of 10 mM phosphate buffer pH 8.0, and an aliquot of this mixture was treated with an equal volume OPT solution (1 mg/ml). After standing at ambient temperature for 15 min, the fluorescence of the solution was measured on a fluorometer set at an emission wavelength of 420 nm and an excitation wavelength of 350 nm. For the assay of GSSG, an aliquot of liver homogenate in 25% metaphosphoric acid (or plasma) was mixed with 0.04 M NEM in a 10:4 ratio. After 30 min, the sample was diluted with 0.1 N sodium hydroxide in a ratio of 1:6, and an aliquot of this dilution was treated with an equal volume of OPT. The fluorescence of the dilution was read on a fluorometer as described for GSH. The concentrations of GSH and GSSG in the sample were derived by reference to calibration curves of GSH and GSSG prepared from serial dilutions of GSH and GSSG stock solutions that were treated in an identical manner as the lung homogenate or plasma sample. The results were reported as µM/g of tissue in the case of liver samples and as µg/ml in the case of plasma samples.

### Assay of GR activity

The GR activity was measured based on the rate of oxidation of NADPH to NADP^+^, a process that is accompanied by a decrease in absorbance at 340 nm [35]. A reaction mixture for this assay was prepared by diluting a portion of liver homogenate with 10 mM phosphate buffer pH 7.4 in a 1:10 ratio, adding 50 µl of 20 mM GSSG, and incubating the mixture at 37°C for 3 min. The reaction was started by adding 50 µl of 1.5 mM NADPH in 01% sodium bicarbonate, and monitoring the consumption of NADPH at 340 nm for 5 min. The activity of GR was calculated using an extinction coefficient for NADPH of 6.22 mM^-1^•cm^-1^ and was expressed as µM of NADPH consumed/min/mg of protein.

### Assay of GCS activity

The GCS activity was measured by a spectrophotometric method in which the rate of formation of ADP generated in the GCS-catalyzed reaction was measured by coupling it to LDH-PK system [36]. The assay medium consisted of 100 µl 100 M Tris-HCl buffer pH 8.2, 100 µl 200 mM magnesium chloride, 100 µl 1.5 M potassium chloride, 100 µl L-glutamic acid, 100 µl 100 mM L-cysteine, 50 µl 100 mM ATP, 133 µl 1.5 mM NADH, 100 µl 20 mM phosphoenolpyruvate, 50 µl 40 U/ml PK and 2 µl 1KU/ml LDH. After initiating the reaction by the addition of 100 µl of sample (plasma or liver homogenate) and incubating at 37°C for 3-4 min, the change in absorbance at 340 nm, due to the oxidation of NADH, was monitored for 1 min. The GCS activity of the sample was expressed in U/min/mg of protein, where one unit is defined as the amount of enzyme catalyzing the consumption of 1 µM of NADH/min/mg of protein under the assay conditions used.

### Assay of GST activity

The assay of the GST activity was based upon the GST-catalyzed reaction between GSH and CDNB, a GST substrate, to produce a colored dinitrophenyl thioether that can be measured on a spectrophotometer set at 340 nm [37]. The reaction mixture contained 100 µl of liver homogenate (or plasma sample), 1.7 ml of phosphate buffer pH 7.4, 100 µl of 20 mM GSH, and 100 µl of 20 mM CDNB. The rate of formation of a CDNB-GSH conjugate at 25°C was monitored on a spectrophotometer at 340 nm for 5 min. The GST activity, defined as the amount of enzyme producing 1 µmol of CDNB-GSH conjugate/min under the conditions of the assay, was calculated using an extinction coefficient for CDNB of 9.6 mM^-1^•cm^-1^. The results were expressed in U/min/mg of protein.

### Statistical analysis

The experimental results are expressed as mean ± standard error of the mean (SEM) for n = 6. Differences between the control and the various treatment groups were determined by Student’s t-test followed by one-way analysis of variance (ANOVA) and Newman-Keuls multiple-range test. A p value <0.05 was taken as an indication of a statistically significant difference.

## Results

### The effect of APAP, alone and in combination with a sulfur-containing compound, on enzymatic indices of hepatocellular damage

**Figure 1:**
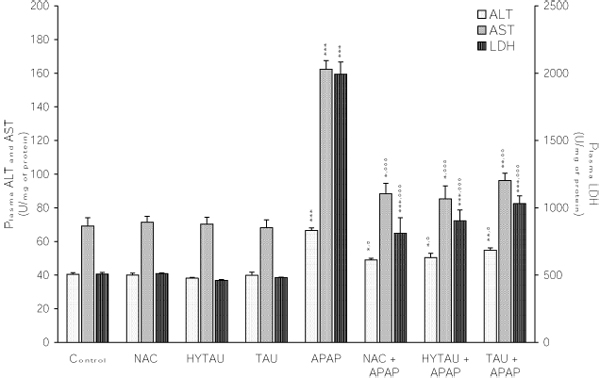
**Attenuation by NAC, HYTAU and TAU of APAP-induced elevation of the plasma ALT, AST and LDH activities.** Differences were significant at *p<0.05, **p<0.01 and ***p<0.001 vs. control; and at °p<0.05, °°p<0.01 and °°°p<0.001 vs. APAP. Each bar represents the mean ± S.E.M. for n = 6.

**Figure 2:**
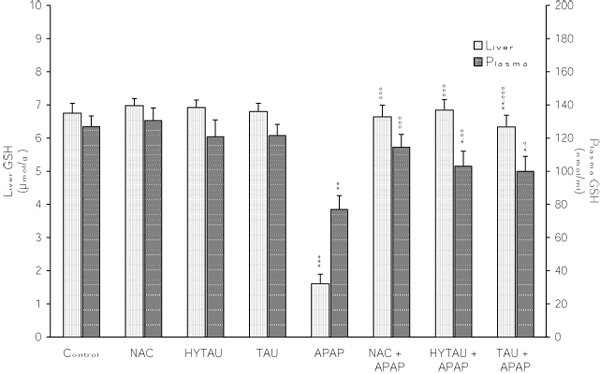
**Attenuation by NAC, HYTAU and TAU of APAP-induced depletion of the plasma and liver GSH.** Differences were significant at *p<0.05, **p<0.01 and ***p< 0.001 vs. control; and at °p<0.05, °°p<0.01 and °°°p<0.001 vs. APAP. Each bar represents the mean ± S.E.M. for n = 6.

**Figure 3:**
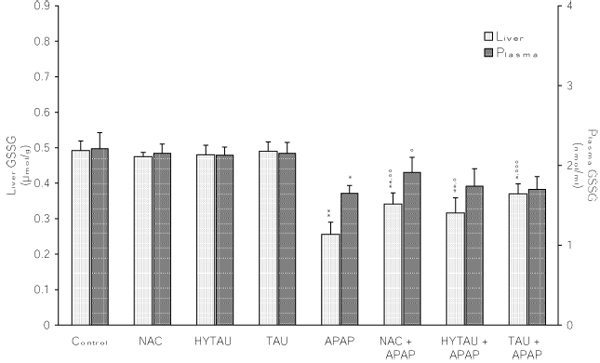
**Attenuation by NAC, HYTAU and TAU of APAP-induced depletion of the plasma and liver GSSG.** Differences were significant at *p<0.05 and **p<0.01 vs. control; and at °p<0.05, °°p<0.01 and °°°p<0.001 vs. APAP. Each bar represents the mean ± S.E.M. for n = 6.

The occurrence of hepatocellular damage induced by a toxic (800 mg/kg) dose of APAP and the influence of a pretreatment with a sulfur-containing compound was investigated by measuring the leakage of hepatic ALT, AST and LDH into the circulation. As shown in Figure 1, APAP elevated the plasma ALT, AST and LDH by 64%, 135% and 292% above control, respectively (all differences at p<0.001 vs. control). Regardless of the enzyme tested, all the test compounds demonstrated a protective effect against these elevations. For example, the plasma ALT rose to only 24%, 35% and 21% in the presence of HYTAU, TAU and NAC, respectively (all differences at p<0.05 vs. control) (Figure 1). Likewise, the elevation in plasma AST were limited to 23% (p<0.05) by HYTAU, to 39% by TAU (p<0.01) and to 34% (p<0.01) by NAC above control (Figure 2); and that of LDH to 78% by HYTAU, to 103% by TAU and to 60% by NAC (all differences at p<0.001 vs. APAP) (Figure 3).

### The effect of APAP, alone and in combination with a sulfur-containing compound, on the liver GSH, GSSG and GSH/GSSG ratio

**Figure 4:**
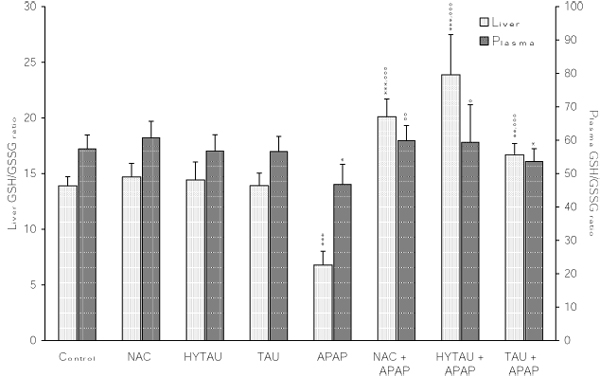
**Attenuation by NAC, HYTAU and TAU of APAP-induced lowering of the plasma and liver GSH/GSSG ratio.** Differences were significant at *p<0.05, **p<0.01 and ***p<0.001 vs. control; and at °p<0.05, °°p<0.01 and °°°p<0.001 vs. APAP. Each bar represents the mean ± S.E.M. for n = 6.

As seen in Figure 2, a toxic dose of APAP lowered the liver GSH by 76% of the control value (p<0.001). While a pretreatment with HYTAU reversed this effect (1% above control), one with TAU (6% decrease) or NAC (only 2% decrease) resulted in significant attenuation. APAP also lowered the liver GSSG content by 47% of the control value (p<0.001). This decrease was limited to 35% (p<0.01) by HYTAU, to 22% (p<0.05) by TAU, and to 31% (P<0.01) by NAC relative to control (Figure 3). As a result of the greater lowering of the GSH content relative to that of GSSG by APAP, the corresponding GSH/GSSG ratio was found to be significantly below the control value (by 54%, p<0.001) (Figure 4). All the pretreatment compounds were able to raise the ratio to a value above control, with HYTAU (74% increase, p<0.001) being much more effective than either TAU (21% increase, p<0.05) or NAC (46% increase, p<0.001) (Figure 4).

### The effect of APAP, alone and in combination with a sulfur-containing compound, on the plasma GSH, GSSG and GSH/GSSG ratio

A 39% (p<0.01) reduction in plasma GSH was observed following a treatment with a toxic dose of APAP. This reduction amounted to only 19% after a pretreatment with HTAU (p<0.05) and to only 7% in the presence of NAC, but was still high (by 28%, p<0.01) in the presence of TAU compared to the control value (Figure 2).  APAP lowered the plasma GSSG by 25% of the control value (p<0.05); with the change being affected by a sulfur-containing compound to different extents (22% decrease, p<0.05, with HYTAU; 2% decrease with TAU; 14% decrease with NAC) (Figure 3). APAP lowered the plasma GSH/GSSG ratio by 19% of the control value (p<0.05). The value of this ratio was increased to above control by HYTAU (by 3%) and NAC (by 4%) but remained below control (by 26%, p<0.01) in the presence of TAU (Figure 4).

### The effect of APAP, alone and in combination with a sulfur-containing compound, on the liver activities of GR, GST and GCS

**Figure 5:**
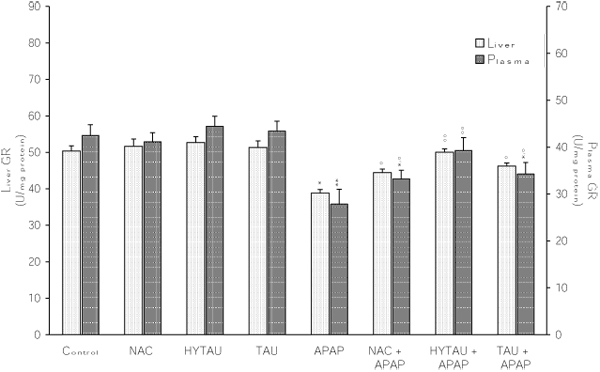
**Attenuation by NAC, HYTAU and TAU of APAP-induced reduction in plasma and liver GR activity.** Differences were significant at *p<0.05 and **p<0.01 vs. control; and at °p<0.05 and °°p<0.01 vs. APAP. Each bar represents the mean ± S.E.M. for n = 6.

**Figure 6:**
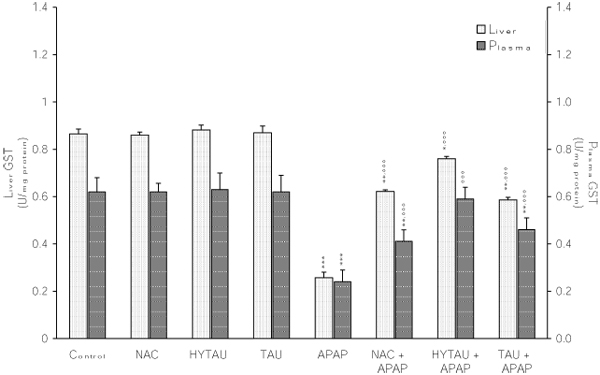
**Attenuation by NAC, HYTAU and TAU of APAP-induced reduction in plasma and liver GST activity.** Differences were significant at *p<0.05, **p<0.01 and ***p< 0.001 vs. control; and at °°°p<0.001 vs. APAP. Each bar represents the mean ± S.E.M. for n = 6.

**Figure 7:**
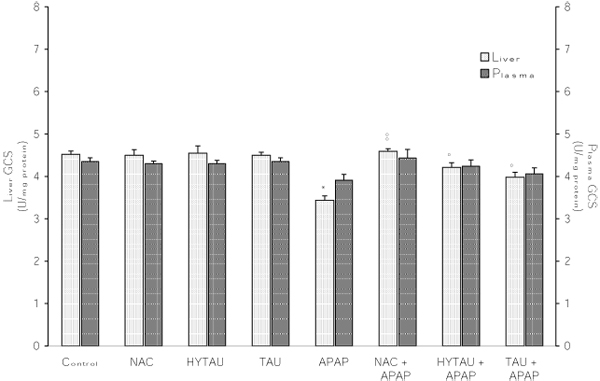
**Attenuation by NAC, HYTAU and TAU of APAP-induced reduction in plasma and liver GCS activity.** Differences were significant at *p<0.05 vs. control; and at °p<0.05 and °°p<0.01 vs. APAP. Each bar represents the mean ± S.E.M. for n = 6.

As shown in Figures 5, 6, 7, a toxic dose of APAP exerted a lowering effect on the hepatic activities of GR, GST and GCS. In the case of GR, the activity was reduced by 23% (p<0.05) of the control value (Figure 5). However, this effect was virtually reversed by HYTAU (only 1% decrease) and effectively attenuated by TAU (only 8% decrease) and NAC (only 12% decrease). From the results presented in Figure 6, it can be seen that APAP lowered the liver GST activity by 70% of the control value (p<0.001), and that this effect was effectively reduced by HYTAU (only 12% decrease), TAU (only 31% decrease, p<0.01) and NAC (only 28% decrease, p<0.01). Similarly, while APAP lowered the liver GCS activity by 24% of the control value (p<0.05), a pretreatment with either HYTAU (only 7% decrease) or TAU (only 12% decrease) led to significant attenuation and one with NAC (1.5% above control) to complete reversal of the APAP effect (Figure 7).

### The effect of APAP, alone and in combination with a sulfur-containing compound, on the plasma activities of GR, GST and GCS

The results summarized in Figure 5, 6, 7 indicate that a toxic dose of APAP lowered the plasma activities of GR, GST and GCS, respectively, and that all the test compounds offered different degrees of protection against such losses. Thus, while a treatment with APAP reduced the GR activity by ~34% of control (p<0.01), a combined treatment with HYTAU (only 8% loss), TAU (~20% loss, p<0.05) or NAC (22% loss, p<0.05) led to a significant attenuation when compared to control (Figure 5). Similarly, while APAP lowered the plasma GST activity by 61% of control (p<0.001), a pretreatment with HYTAU (5% loss), TAU (26% loss, p<0.01) or NAC (34% loss, p<0.01) reduced the effect of APAP to different extents (Figure 6).  In terms of the GCS activity, there was an insignificant (10%) decrease after a treatment with APAP, and either reduction of the effect after a co-treatment with HYTAU (3% loss) or TAU (7% loss) or a reversal of the effect (2% raise) after a co-treatment with NAC (Figure 7).

### The effect of APAP, alone and in combination with a sulfur-containing compound, on the liver and plasma MDA

**Figure 8:**
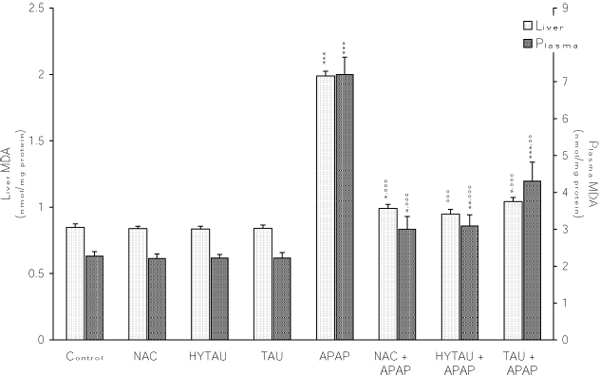
**Attenuation by NAC, HYTAU and TAU of APAP-induced increase in plasma and liver MDA.** Differences were significant at *p<0.05, **p<0.01 and ***p< 0.001 vs. control; and at °°p<0.01 and °°°p<0.001 vs. APAP. Each bar represents the mean ± S.E.M. for n = 6.

From the results presented in Figure 8, it is evident that APAP promoted LPO by increasing the levels of MDA both in the liver (134%) and plasma (by 217%) above control values (p<0.001 for both). The same Figure also indicates that a pretreatment with HYTAU (12% increase), TAU (22% increase, p<0.05) or NAC (16% increase, p<0.05) was effective in counteracting the altering action of APAP on the liver MDA (p<0.001 vs. APAP). Likewise, the increase in the plasma MDA induced by APAP was reduced to 32% by NAC (p<0.01), to 36% by HYTAU (p<0.01), and to 90% by TAU (p<0.001) relative to the control value (Figure 8).

## Discussion

This study has compared NAC, TAU and HYTAU for their ability to protect the liver against the oxidative stress and hepatocellular injury that follows a supratherapeutic dose of APAP. All the experiments were conducted with male Sprague-Dawley rats since this animal model was previously found appropriate for assessing the role of TAU in preventing hepatic injury by a toxic dose of APAP [29]. The dose (2.4 mmol/kg) and the route and timing of the administration of the various test compounds were based on the results of earlier studies from this laboratory and which found TAU to exhibit antioxidant properties capable of protecting erythrocytes against the deleterious effects of oxidative stress, including LPO, GSH depletion, and enzyme inactivation [30,31]. Protection by a sulfur-containing compound against hepatotoxicity by APAP was gauged on the basis of the increases in the plasma activities of enzymes serving as indices of hepatic injury (i.e., ALT, AST, LDH) and on the extent of oxidative stress, inferred from the levels of MDA, GSH and GSSH and the activities of enzymes relevant to GSH redox cycling (GR), conjugating transfer to appropriate substrates (GST) and synthesis (GCS) in both the plasma and liver.

NAC is regarded as the antidote of choice for treating APAP overdoses. The most accepted explanation for the protective actions of this N-substituted amino acid derivative is that it serves as a source of L-cysteine for GSH synthesis and, hence, it can facilitate the detoxification of NAPQI before this reactive metabolite can initiate hepatic injury [17,38]. However, NAC is only protective as long as a viable cysteine-driven synthesis of GSH is operative [25] since it is ineffective when the hepatic store of GSH is artificially depleted by a treatment with buthionine sulfoximine, an inhibitor of GCS [18,39]. A more recent proposal, put forth to account for the protective mechanism of NAC in APAP overdoses, is that this cysteine analog acts as a scavenger of ROS and OONO^-^ and that it supports mitochondrial energy metabolism [40].

Since TAU, an end product of the metabolism of cysteine through the cysteinesulfinic acid pathway [41], is a poor scavenger of oxygen-derived free radicals [42], it antioxidant effects on the liver are probably exerted indirectly, possibly by preventing LPO [29], by suppressing ROS formation [43,44] or by protecting mechanisms that replenish the intracellular stores of GSH during oxidative disturbances [45,46]. Alternatively, TAU may attenuate oxidative stress and, thereby, prevent the loss of GSH, by increasing the activities of antioxidant enzymes such as CAT and GPX to scavenge cytotoxic ROS [47,48].

HYTAU, the sulfinate analog of TAU and the immediate biological precursor of TAU, has been compared to TAU as a scavenger of free radicals, H_2_O_2_ and HClO in cell-free ROS generating systems [26,43] and in plasma free hemocytes [49]. While HYTAU was able to scavenge HClO and HO•, it was unreactive towards the O_2_^-•^ and H_2_O_2_[26,43,49]. In contrast, TAU was found to be a poor scavenger of free radicals and H_2_O_2_ and to readily bind to HClO [26]. Still, other studies have found further differences between these sulfur compounds, including protection by HYTAU, but not by TAU, against ONOO^-^-related cell injury possibly because of the oxidability of its sulfinic group to sulfonate by ONOO^-^[50]. Moreover, the addition of HYTAU to a sperm preparation was reported to decrease H_2_O_2_-induce ROS formation and to protect against H_2_O_2_-induced DNA damage [51].

In spite of the differences in structural features and in intrinsic antioxidant characteristics among the three test compounds evaluated here, they were all found to offer significant protection against the hepatotoxic effects of APAP. However, and except for a few isolated instances, HYTAU and NAC were generally more protective than TAU. In addition to contributing to the maintenance of the membrane integrity of hepatocytes, as inferred from the lower plasma activities of ALT, AST and LDH, serving as markers of liver damage, these compounds were also able to markedly reduce the production of MDA, an indicator of LPO, and to return the intracellular levels of GSH to values that were equal to (HYTAU) or just below (NAC, TAU) the control value. Likewise, all three test compounds were able to attenuate the lowering action of APAP on the liver GSSG content by about one-half (TAU) or better (HYTAU, NAC) but not to the point of preventing the reversal of the hepatic GSH/GSSG ratio seen with APPA alone, namely from below to above the control value. Surprisingly, HYTAU showed a greater ability to elevate the GSH/GSSG ratio than NAC even though HYTAU is not a precursor of GSH which is the case of NAC. On the other hand, a toxic dose of APAP lowered the hepatic activities of those enzymes involved either in de novo synthesis of GSH (i.e., GCS), GSH nucleophilic addition to substrates with electrophilic functional groups (i.e., GST) or in the redox cycling of GSH and GSSG (i.e., GR). The present results indicate that although APAP can negatively affect the activities of these hepatic enzymes, the extent of its effects will vary according to the enzyme, in all likelihood because of the known differences in sensitivity of GSH-related enzymes to a given oxidizing agent [52]. Thus, APAP had a profound effect on GST (70% reduction) and a rather moderate one (~24% reduction) on both GCS and GR. Within a narrow range of potencies, HYTAU was somewhat more effective than NAC in curtailing the changes in GR and GST caused by APAP; and NAC was the only treatment compound to raise the GCS activity to baseline values. In contrast, TAU was equipotent to HYTAU in preserving the GR activity, about equipotent to NAC in preserving the GST activity, and weaker than either HYTAU or NAC in protecting the GCS activity.

GR is an enzyme that plays a critical role in oxidative stress by APAP since a decrease in its activity will lead to interruption of the cycling between GSSG and GSH and, thus, to GSH shortage. Although the impairment of GR activity by APAP is not well understood, at least two hypothesis have been put forth to explain this occurrence, one invoking a direct action of ROS or toxic aldehydes and another ascribing the effect to the conjugate between NAPQI and GSH that forms in the presence of GST [13]. The noted difference in inhibitory action by APAP on GSH-related enzymes is in close agreement with the results of in vitro experiments with cultured human cells and in which the exposure of enzymes germane to GSH utilization and redox cycling to H_2_O_2_ or to different organic peroxides revealed marked differences in susceptibility to inactivation. Indeed, while glutathione peroxidase (GPX), a SH-requiring enzyme participating  in peroxide elimination, was found to be highly susceptibility to inhibition by peroxides, GR and GST remained unaltered [52,53]. Moreover, the acute administration of a single 375 mg/kg of APAP to mice was reported to decrease the total GSH content, GSH/GSSG ratio and activities of selenium and non-selenium-dependent GPX activities, and to increase O_2_^-•^ production. Due to the inhibition of GPX, this study concluded that hepatic cell injury was the result of an increase in the steady state level of H_2_O_2_ and hydroperoxides [54]. While this suggestion implies that oxidative stress is determining factor of GSH depletion, there is also evidence to support the opposite order of events, namely that ROS production follows the depletion of GSH [55].

Protection by NAC, HYTAU and TAU against APAP-induced liver injury is also a reflection of their ability to prevent the loss of GCS activity to a toxic dose of APAP, an enzyme that plays a crucial role in protecting the liver against hepatotoxic compounds by regulating the de novo synthesis of GSH. The importance of this role for GCS has been experimentally verified through the use γ-GCS knockdown rats [56] or a specific inhibitor of GCS activity such as buthionine sulfoximine [18] and in which hepatotoxicity by APAP was more extensive than in normal or uninhibited animals. Conversely, transgenic mice with enhanced GCS activity were found resistant to APAP-induced liver injury [57].

Regarding the alterations in biochemical parameters due to a high dose of APAP and detected in plasma samples, it is apparent that while they all closely parallel the changes observed in the liver, they were, in all but one instance, of a lesser magnitude. The only notable exception was the level of MDA which was almost 2-fold greater in the plasma than in the liver. In terms of the test compounds, and in common with the findings for the liver samples, HYTAU was again somewhat more potent than NAC and TAU was again the least potent in attenuating APAP-induced biochemical alterations.

In conclusion, the present study has determined that when NAC, HYTAU or TAU are administered to rats in equal doses and as a pretreatment to a toxic dose of APAP, they can equally attenuate the hepatocellular damage, oxidative stress, and alterations in GSH redox cycling, utilization and transfer caused by APAP. Although HYTAU and TAU do not play a role in the biosynthesis of hepatic of GSH, as NAC does, they are, unexpectedly, equipotent to NAC in maintaining a normal store of GSH and a normal GSH/GSSH ratio in the liver. The protective actions of the tests compounds, based on the magnitude of their actions on the liver and plasma alterations brought about by APAP decreased in the approximate order HYTAU>NAC>TAU. Also, the present results clearly suggest that the antioxidant actions demonstrated by these sulfur-containing compounds in an animal model of APAP toxicity would not have been predicted from the results for antioxidant activity gathered using cell-free in vitro systems generating free radicals or including a peroxide compound.

## Abbreviations

PBS: phosphate buffered saline; TBARS: thiobarbituric acid reactive substances; TCA: trichloroacetic acid; TBA: thiobarbituric acid; HCl: hydrochloric acid; TEP: 1,1,3,3-tetraethoxypropane; GSH: reduced glutathione; GSSG: oxidized glutathione; OPT: *o*-phthalaldehyde; NEM: N-ethylmaleimide; LDH: lactate dehydrogenase; PK: pyruvate kinase; CDNB: 1-chloro-2,4-dinitrobenzene.

## Competing interests

The authors declare that they have no competing interests.

## Authors’ contributions

MA carried out all experimental work on live animals, performed all the biochemical assays and statistical analyses, prepared the figures, helped with the collection of bibliographical information, and made editorial comments to the article. CAL conceived the project and guided its development, assembled, organized and interpreted the experimental data, and reviewed the pertinent scientific literature.

## References

[B1] Henderson CJ Wolf CR Kitteringham N Powell H Otto D Park BK Increased resistance to acetaminophen hepatotoxicity in mice lacking glutathione S-transferase Pi. Proc Nat Acad Sci USA 2000 97 12741 12745 10.1073/pnas.220176997 11058152 PMC18834

[B2] Dahlin DC Miwa GT Lu AYH Nelson SD N-Acetyl-p-benzoquinone imine; a cytochrome P-450-mediated oxidation product of acetaminophen Proc Nat Acad Sci U S A 1984 81 1327 1331 10.1073/pnas.81.5.1327 PMC344826 6424115

[B3] Hazelton GA Hjelle JJ Klaassen CD Effects of cysteine pro-drugs on acetaminophen-induced hepatotoxicity J Pharmacol Exp Ther 1986 237 341 349 3958971

[B4] Lauterburg BH Mitchell JR Toxic doses of acetaminophen suppress hepatic glutathione synthesis in rats Hepatology 1982 2 8 12 10.1002/hep.1840020103 7054070

[B5] Yonamine M Aniya Y Yokomakura T Koyama T Nagamine T Nakanishi H Acetaminophen-derived activation of liver microsomal glutathione S-transferase of rats Jpn J Pharmacol 1996 72 175 181 10.1254/jjp.72.175 8912918

[B6] James LP Mayeux PR Hinson JA Acetaminophen-induced hepatotoxicity Drug Metab Dispos 2003 31 1499 1506 10.1124/dmd.31.12.1499 14625346

[B7] James LP McCullough SS Lamps LW Hinson JA Effect of N-acetylcysteine on acetaminophen toxicity in mice; relationship to reactive nitrogen and cytokine formation Toxicol Sci 2003 75 458 467 10.1093/toxsci/kfg181 12883092

[B8] Reid AB Kurten RC McCullough SS Brock RW Hinson JA Mechanisms of acetaminophen-induced hepatotoxicity: role of oxidative stress and mitochondrial permeability transition in freshly isolated muse hepatocytes. J Pharmacol Exp Ther 2005 312 509 516 10.1124/jpet.104.075945 15466245

[B9] Nelson SD Mechanism of the formation and disposition of reactive metabolites that can cause acute liver injury Drug Metab Rev 1995 27 147 177 10.3109/03602539509029821 7641574

[B10] Bessems JGM Vermeulen NPE Paracetamol (acetaminophen)-induced toxicity: molecular and biochemical mechanisms, analogues and protective approaches Crit Rev Toxicol 2001 31 55 138 10.1080/20014091111677 11215692

[B11] Adamson GM Harman AW Oxidative stress in cultured hepatocytes exposed to acetaminophen Biochem Pharmacol 1993 45 2289 2294 10.1016/0006-2952(93)90201-7 8517869

[B12] Jaeschke H Glutathione disulfide formation and oxidant stress during acetaminophen-induced hepatotoxicity in mice in vivo: the protective effect of allopurinol J Pharmacol Exp Ther 1990 255 935 941 2262912

[B13] Roušar T Pařík P Kučera O Bartoš M Cervinková Z Glutathione reductase is inhibited by acetaminophen-glutathione conjugate in vitro Physiol Res 2009 58 239 246 19537930 10.33549/physiolres.931744

[B14] O'Brien PJ Slaughter MR Swain A Birmingham JM Greenhill RW Elcok F Bugelski PJ Repeated acetaminophen dosing in rats: adaptation of hepatic antioxidant system Hum Exp Toxicol 2000 19 277 283 10.1191/096032700678815918 10918522

[B15] Jaeschke H Bajt ML Intracellular signaling mechanisms of acetaminophen-induced cell death Toxicol Sci 2006 89 31 41 10.1093/toxsci/kfi336 16177235

[B16] Crankshaw DL Berkeley LI Cohen JF SHirota FN Nagasawa HT Double-prodrugs of L-cysteine: differential protection against acetaminophen-induced hepatotoxicity in mice J Biochem Mol Toxicol 2002 16 235 244 10.1002/jbt.10044 12439865

[B17] Lauterberg BH Corcoran GB Mitchell JR Mechanism of action of N-acetyl-L-cysteine in the protection against the hepatotoxicity of acetaminophen in rats in vivo J Clin Invest 1983 71 980 991 10.1172/JCI110853 6833497 PMC436956

[B18] Miners JO Drew R Birkett DJ Mechanism of action of paracetamol protective agents in mice in vivo Biochem Pharmacol 1984 33 2995 3000 10.1016/0006-2952(84)90599-9 6487352

[B19] Valentovic M Terneus M Harmon RC Carpenter AB S-Adenosylmethionine (SAMe) attenuates acetaminophen hepatotoxicity in C57BL/6 mice Toxicol Lett 2004 154 165 174 10.1016/j.toxlet.2004.07.010 15501608

[B20] Fairhurst S Barber DJ Clark B Horton AA Studies on paracetamol-induced lipid peroxidation. Toxicology 1982 23 249 59 10.1016/0300-483X(82)90102-0 7112597

[B21] Knight TR Fariss MW Farhood A Jaeschke H Role of lipid peroxidation as a mechanism of liver injury after acetaminophen overdose in mice Toxicol Sci 2003 76 229 236 10.1093/toxsci/kfg220 12944590

[B22] Lake BG Harris RA Phillips JC Gangolli SD Studies on the effects of L-ascorbic acid on acetaminophen-induced hepatotoxicity: 1. Inhibition of the covalent binding of acetaminophen metabolites to hepatic microsomes in vitro Toxicol Appl Pharmacol 1981 60 229 240 10.1016/0041-008X(91)90227-6 7281186

[B23] Nakae D Yamamoto Y Yoshiji T Kinugasa T Maruyama H Farber JL Konishi Y Liposome-encapsulated superoxide dismutase prevents liver necrosis induced by acetaminophen. Am J Pathol 1990 136 787 795 2158237 PMC1877636

[B24] Sakaida I Kayano K Wasaki S Nagatomi A Matsumura Y Okita K Protection against acetaminophen-induced liver injury in vivo by an iron chelator, deferoxamine Scand J Gastroenterol 1995 30 61 67 10.3109/00365529509093237 7701253

[B25] Corcoran GB Wong BK Role of glutathione in prevention of acetaminophen-induced hepatotoxicity by N-acetyl-L-cysteine in vivo: studies with N-acetyl-D-cysteine in mice J Pharmacol Exp Ther 1986 238 54 61 3723405

[B26] Aruoma OI Halliwell B Hoey BM Butler J The antioxidant action of N-acetylcysteine: its reaction with hydrogen peroxide, hydroxyl radical, superoxide, and hypochlorous acid. Free Radic Biol Med 1989 6 593 597 10.1016/0891-5849(89)90066-X 2546864

[B27] Çay A Alver A Küçük M Isik O Selçuk Eminağaoğlu M Caner Karahan S Değer O The effects of N-acetylcysteine on antioxidant enzyme activities in experimental testicular torsion. J Surg Res 2006 131 199 203 10.1016/j.jss.2005.11.572 16412470

[B28] Dekhuijzen PNR Antioxidant properties of N-acetylcysteine: their relevance in relation to chronic obstructive pulmonary disease Eur Respir J 2004 23 629 636 10.1183/09031936.04.00016804 15083766

[B29] Waters E Wang JH Redmond HP Wu QD Kay E Bouchier-Hayes D Role of taurine in preventing acetaminophen-induced hepatic injury in the rat Am J Physiol Gastrointest Liver Physiol 2001 280 G1274 G1279 11352821 10.1152/ajpgi.2001.280.6.G1274

[B30] Gossai D Lau-Cam C The effects of taurine, taurine homologs and hypotaurine on cell membrane and antioxidative system alterations caused by type 2 diabetes in rat erythrocytes Adv Exp Med Biol 2009 643 85 93 full_text 19239167 10.1007/978-0-387-75681-3_37

[B31] Pokhrel PK Lau-Cam CA In vitro and in vivo effects of taurine and structurally related sulfur-containing compounds against phenylhydrazine-induced oxidative damage to erythrocytes. Adv Exp Med Biol 2000 483 503 522 full_text 11787637 10.1007/0-306-46838-7_56

[B32] Green TR Fellman JH Eicher AL Pratt KL Antioxidant role and subcellular location of hypotaurine and taurine in human neutrophils Biochim Biophys Acta 1991 1073 91 97 1846756 10.1016/0304-4165(91)90187-l

[B33] Bugue JA Aust SD Microsomal lipid peroxidation Methods Enzymol 1978 52 302 310 full_text 672633 10.1016/s0076-6879(78)52032-6

[B34] Hissin JP Hilf R A fluorometric method for determination of oxidized and reduced glutathione in tissues Anal Biochem 1976 74 214 226 10.1016/0003-2697(76)90326-2 962076

[B35] Zhou W Freed CR DJ-1 up-regulates glutathione synthesis during oxidative stress and inhibits A53T α-synuclein toxicity. J Biol Chem 2005 280 43150 43158 10.1074/jbc.M507124200 16227205

[B36] Wheeler CR Salzman JA Elsayed NM Omaye ST Korte DW Jr Automated assays for superoxide dismutase, catalase, glutathione peroxidase, and glutathione reductase activity. Anal Biochem 1990 184 193 199 10.1016/0003-2697(90)90668-Y 2327564

[B37] Habig WH Pabst MJ Jakoby WB Glutathione S-transferases. The first enzymatic step in mercapturic acid formation. J Biol Chem 1974 249 7130 7139 4436300

[B38] Corcoran GB Todd EL Racz WJ Hughes H Smith CV Mitchell JR Effects of N-acetylcysteine on acetaminophen covalent binding and hepatic necrosis in mice. J Pharmacol Exp Ther 1985 232 864 872 3973835

[B39] Drew R Miners JO The effects of buthionine sulphoximine (BSO) on glutathione depletion. Biochem Pharmacol 1984 33 2989 2994 10.1016/0006-2952(84)90598-7 6148944

[B40] Saito C Zwingmann C Jaeschke H Novel mechanisms of protection against acetaminophen hepatotoxicity in mice by glutathione and N-acetylcysteine Hepatology 2010 51 246 254 19821517 10.1002/hep.23267 PMC2977522

[B41] Weinstein CL Haschmeyer RH Griffith OW In vivo studies of cysteine metabolism. Use of D-cysteinesulfinate, a novel cysteinesulfinate decarboxylase inhibitor, to probe taurine and pyruvate synthesis. J Biol Chem 1988 263 16568 16579 3182803

[B42] Shi X Flynn DC Porter DW Leonard SS Vallyathan V Castranova V Efficacy of taurine based compounds as hydroxyl radical scavengers in silica induced peroxidation. Ann Clin Lab Sci 1997 27 365 374 9303176

[B43] di Wu Q Wang JH Fennessy F Redmond HP Boucher-Hayes D Taurine prevents high-glucose-induced human vascular endothelial cell apoptosis Am J Physiol Cell Physiol 1999 277 C1229 C1238 10.1152/ajpcell.1999.277.6.C1229 10600775

[B44] Ito T Muraoka S Takahashi K Fujio Y Schaffer SW Azuma J Beneficial effects of taurine against doxorubicin-induced cardiotoxicity in mice Adv Exp Med Biol 2009 643 65 74 full_text 19239137 10.1007/978-0-387-75681-3_7

[B45] Derlacz RA Sliwinska M Piekutowska A Winiarska K Drozak J Bryla J Melatonin is more effective than taurine and 5-hydroxytryptophan against hyperglycemia-induced kidney cortex tubules injury J Pineal Res 2007 42 203 209 10.1111/j.1600-079X.2006.00405.x 17286753

[B46] Yu J Kim AK Effect of taurine on antioxidant enzymes system in B16F10 melanoma cells Adv Exp Med Biol 2009 643 491 499 full_text 19239181 10.1007/978-0-387-75681-3_51

[B47] Samipillai SS Jagadeesan G Protective role of taurine against mercuric chloride intoxicated rats Rec Res Sci Technol 2009 1 081 087

[B48] Pushpakiran G Mahalakshmi K Anurandha CV Protective effects of taurine on glutathione and glutathione-dependent enzymes in ethanol-fed rats Pharmazie 2004 59 869 872 15587589

[B49] Hahn UK Bender RC Bayne CJ Killing of Schistosoma mansoni sporocysts by hemocytes from resistant Biomphalaria glabrata: role of reactive oxygen species. J Parasitol 2001 87 292 299 2001 11318558 10.1645/0022-3395(2001)087[0292:KOSMSB]2.0.CO;2

[B50] Fontana M Duprè S Pecci L The reactivity of hypotaurine and cysteine sulfinic acid with peroxynitrite Adv Exp Med Biol 2006 583 15 24 full_text 17153585 10.1007/978-0-387-33504-9_2

[B51] Donnelly ET McClure N Lewis SEM Glutathione and hypotaurine in vitro: effects on human sperm motility, DNA integrity and production of reactive oxygen species. Mutagenesis 2000 15 61 68 10.1093/mutage/15.1.61 10640532

[B52] Ochi T Effects of an organic hydroperoxide on the activity of antioxidant enzymes in cultured mammalian cells Toxicology 1990 61 229 239 10.1016/0300-483X(90)90173-E 2330596

[B53] Vessey DA Lee KH Inactivation of enzymes of the glutathione antioxidant system by treatment of cultured human keratinocytes with peroxides J Invest Dermatol 1993 100 829 833 10.1111/1523-1747.ep12476735 8496623

[B54] Arnaiz SL Llesuy S Cutrin JC Boveris A Oxidative stress by acute acetaminophen administration in mouse liver. Free Radic Biol Med 1995 19 303 310 10.1016/0891-5849(95)00023-Q 7557544

[B55] Bajt ML Knight TR Lemasters JJ Jaeschke H Acetaminophen-induced oxidant stress and cell injury in cultured mouse hepatocytes: protection by N-acetylcysteine. Toxicol Sci 2004 80 343 349 10.1093/toxsci/kfh151 15115886

[B56] Akai S Hosomi H Minami K Tsuneyama K Katoh M Nakajima M Yokoi T Knock down of γ-glutamylcysteine synthetase in rat causes acetaminophen-induced hepatotoxicity. J Biol Chem 2007 282 23996 24003 10.1074/jbc.M702819200 17573345

[B57] Botta D Shi S White CC Dabrowski MJ Keener CL Srinouanprachanh SL Farin FM Ware CB Ladiges WC Pierce RH Acetaminophen-induced liver injury is attenuated in male glutamate-cysteine ligase transgenic mice J Biol Chem 2006 281 28865 28875 10.1074/jbc.M605143200 16840778

